# Beyond a Universal Cut-Off: [^18^F]FET-PET Metrics for Distinguishing High-Grade Glioma Recurrence from Treatment-Related Changes

**DOI:** 10.3390/brainsci16101085

**Published:** 2026-10-09

**Authors:** Pier Paolo Berti, Alessandro Spimpolo, Leda Lorenzon, Davide Drusiani, Matteo Presa, Luca Zavatto, Paolo Cipriano Cecchi, Davide Costazza, Vania Pirillo, Matteo Bonatti, Francesco Erdini, Nadia Oberhofer, Giuseppe Maria Della Pepa, Francesco Doglietto, Andreas Schwarz, Mohsen Farsad

**Affiliations:** 1 Department of Neurosurgery, Hospital of Bolzano (SABES-ASDAA), Teaching Hospital of Paracelsus Medical University (PMU), 39100 Bolzano-Bozen, Italy; 2 Department of Nuclear Medicine, Hospital of Bolzano (SABES-ASDAA), Teaching Hospital of Paracelsus Medical University (PMU), 39100 Bolzano-Bozen, Italy; 3 Medical Physics Service, Hospital of Bolzano (SABES-ASDAA), Teaching Hospital of Paracelsus Medical University (PMU), 39100 Bolzano-Bozen, Italy; 4 Radiology Service, Hospital of Bolzano (SABES-ASDAA), Teaching Hospital of Paracelsus Medical University (PMU), 39100 Bolzano-Bozen, Italy; 5 Department of Pathology, Hospital of Bolzano (SABES-ASDAA), Teaching Hospital of Paracelsus Medical University (PMU), 39100 Bolzano-Bozen, Italy; 6 Institute of Neurosurgery, Fondazione Policlinico Gemelli, Catholic University, 00168 Rome, Italy

**Keywords:** glioma, positron-emission tomography, neoplasm recurrence, local, [^18^F]FET-PET, amino acid PET, pseudoprogression, treatment-related changes, tumour-to-background ratio

## Abstract

**Highlights:**

**What are the main findings?**

- Static semiquantitative [^18^F]FET-PET parameters (except metabolic tumour volume) showed comparable moderate-to-good accuracy, supporting the overall consistency and reliability of [^18^F]FET-PET in distinguishing recurrence from TRC;
- Among the evaluated metrics, TBRmean achieved the highest overall diagnostic performance (AUC 0.80) and provided higher specificity (75.0%) than TBRmax (64.3%), effectively reducing false-positive interpretations.

**What are the implications of the main findings?**

- TBRmean represents a pragmatic primary quantitative marker in the post-treatment follow-up of HGGs: unlike maximum-based SUV and TBR indices, it combines high diagnostic discrimination and specificity with lower apparent scanner dependence, thereby reducing the risk of misclassifying TRC as recurrence and prompting unnecessary therapeutic escalation;
- Published and guideline-recommended thresholds for [^18^F]FET-PET should be treated as a reference framework requiring local validation and centre-specific calibration before multidisciplinary integration, rather than universal cut-offs.

**Abstract:**

**Background/Objectives:** Differentiating tumour recurrence from treatment-related changes (TRC) after multimodal treatment of high-grade gliomas (HGGs) remains challenging because of the overlap in imaging findings on conventional magnetic resonance. This study compared the diagnostic performance of the main static semiquantitative O-(2-[^18^F]fluoroethyl)-L-tyrosine positron emission tomography ([^18^F]FET-PET) parameters and assessed the diagnostic contribution of qualitative tracer uptake. **Methods:** This retrospective single-centre study included 77 scans from 61 adult patients with previously resected and chemoradiotherapy-treated HGGs undergoing [^18^F]FET-PET for suspected recurrence. Standardised uptake values (SUVs), tumour-to-background ratios (TBRs), and metabolic tumour volume (MTV) were analysed. Recurrence was determined using a composite histopathological and clinical–radiological reference standard. Diagnostic performance was assessed using receiver operating characteristic analysis, Youden-derived cut-offs and DeLong comparisons. Internal validation was performed by using patient-grouped five-fold cross-validation, in which cut-offs were re-derived within each training partition and applied to the held-out partition. **Results:** Forty-nine scans were classified as recurrence and 28 as TRC. All semiquantitative parameters except MTV were significantly higher in recurrent disease after false-discovery-rate correction. TBR mean ratio showed the highest accuracy (AUC, 0.80; cut-off, 1.85; sensitivity, 77.6%; specificity, 75.0%), followed by SUV max (AUC, 0.793). Neither prespecified comparison among TBR parameters was significant. The presence of pathological uptake achieved 100% sensitivity, 42.9% specificity, 100% NPV, and 79.2% accuracy. MTV was non-discriminative (AUC, 0.499). SUV max differed significantly between scanners (*p* = 0.008). **Conclusions:** Static semiquantitative [^18^F]FET-PET parameters, except MTV, showed comparable moderate-to-good performance for distinguishing recurrence from TRC. TBRmean may represent a pragmatic primary marker due to its background normalisation reduction of scanner-related variability. Guideline-recommended thresholds should be regarded as reference values requiring local validation and, when appropriate, centre- and protocol-specific calibration.

## 1. Introduction

The distinction between tumour recurrence and treatment-related changes (TRC) remains one of the most critical and clinically relevant challenges in the follow-up of patients with high-grade gliomas (HGGs). After surgery, radiotherapy, and chemotherapy, new or enlarging contrast-enhancing lesions and perilesional oedema may reflect either viable tumour or treatment-related tissue injury. These conditions frequently present with overlapping clinical manifestations and radiological appearances, making their differentiation particularly complex. Incorrect interpretation may lead either to the premature discontinuation of an effective treatment or to unnecessary therapeutic escalation, including additional surgery, re-irradiation, or systemic therapy. Among TRC, pseudoprogression typically occurs within the first months after chemoradiotherapy and is thought to arise from transient blood–brain barrier disruption, inflammation, and treatment-induced cellular injury rather than viable tumour growth. Radionecrosis, by contrast, generally develops later and represents an often permanent form of radiation-induced tissue damage [1,2,3,4,5]. Both entities may mimic tumour recurrence on conventional magnetic resonance imaging (MRI), with contrast enhancement, mass effect, and surrounding oedema. Advanced MRI techniques, including perfusion-weighted imaging, diffusion-weighted imaging, and magnetic resonance spectroscopy, may improve diagnostic assessment but can provide inconclusive or discordant findings in most cases [4,6,7]. Additional imaging tools are therefore increasingly used to improve diagnostic confidence and support multidisciplinary decision-making.

In this context, O-(2-[^18^F]fluoroethyl)-L-tyrosine positron emission tomography ([^18^F]FET-PET) has assumed an important role in neuro-oncological imaging. [^18^F]FET uptake primarily reflects increased amino acid transport and is relatively independent of blood–brain barrier disruption. Combined with the low physiological uptake of the tracer in normal brain tissue, these properties improve lesion conspicuity and allow [^18^F]FET-PET to provide complementary data when conventional MRI is inconclusive [8,9,10,11]. Rather than its visual interpretation only, its usefulness relies on the additional extraction of semiquantitative and dynamic parameters that may reflect different biological features of the lesion.

Despite its established diagnostic value, uncertainty remains regarding the selection and interpretation of [^18^F]FET-PET-derived parameters in previously treated gliomas. Commonly used static semiquantitative measures include maximum and mean standardised uptake values (SUVmax), maximum and mean tumour-to-background ratios (TBRmax and TBRmean), and metabolic tumour volume (MTV). Dynamic parameters, such as time to peak (TTP) and the slope of the uptake curve, may provide additional information regarding tracer kinetics, yet the relative diagnostic value of individual parameters remains heavily debated. A major challenge stems from the fact that absolute SUV metrics, most notably SUVmax, are deeply influenced by scanner-specific hardware and reconstruction settings. Because SUVmax relies on a single voxel, its absolute value is intrinsically relative, highly susceptible to image noise, and inherently prone to severe institutional variability [12,13,14,15,16,17,18,19]. Although tumour-to-background normalisation (TBR) is widely recommended to mitigate these variations by dividing lesion uptake by normal brain tissue, TBR-derived measures are still not independent of the segmentation pipeline and background volume-of-interest definition. These profound methodological differences limit the direct transferability of published thresholds and challenge the concept of a single universally applicable cutoff.

The primary aim of this study was to systematically compare the diagnostic performance of the main static [^18^F]FET-PET semiquantitative parameters in differentiating tumour recurrence from TRC in previously treated patients with HGG. Specifically, we evaluated SUVmax, SUVmean, SUVpeak, TBRmax, TBRmean, TBR peak and MTV in terms of their discriminative ability, sensitivity, and specificity. Rather than focusing on a single parameter, we sought to determine which measures were most informative within our cohort and whether any observed differences justified their preferential clinical use. Secondary objectives were to evaluate the diagnostic contribution of the qualitative presence or absence of focal [^18^F]FET uptake and to identify cohort-specific decision thresholds for the quantitative parameters. These values were interpreted in the context of previously reported reference ranges [8] and potential methodological variability related to patient selection, scanner characteristics, acquisition protocols, and image analysis. This study therefore aimed not to establish a universally transferable cutoff, but to provide evidence supporting a clinically applicable and locally validated interpretation of static [^18^F]FET-PET findings within the broader clinical and radiological assessment of suspected HGG recurrence.

## 2. Materials and Methods

### 2.1. Population

This retrospective, single-centre study was conducted at Bolzano Hospital (Bolzano/Bozen, Italy). The study population comprised 61 patients who underwent [^18^F]FET-PET between December 2019 and December 2025. Eligible patients were 18–80 years of age with histomolecular confirmed diagnosis of HGG according to the WHO Classification of Tumours of the Central Nervous System. Eligible tumour entities included IDH-wildtype glioblastoma, CNS WHO grade 4; IDH-mutant astrocytoma, CNS WHO grade 3 or 4; and IDH-mutant and 1p/19q-codeleted oligodendroglioma, CNS WHO grade 3.

All patients underwent gross total resection, defined as the absence of residual contrast-enhancing tumour on postoperative MRI performed within 48 h after surgery [20]. Only patients who were evaluated with [^18^F]FET PET/CT after MRI findings suspicious for tumour recurrence at least 3 months after surgery were included. Patients were also required to have completed standard adjuvant chemoradiotherapy, administered either concomitantly or sequentially according to tumour histology and clinical indications. Patients who received alternative adjuvant treatment regimens or were enrolled in experimental therapeutic protocols were excluded.

### 2.2. Surgical Procedure

All patients underwent surgical resection with radical intent, achieving gross total resection. Fluorescence-guided surgery with 5-aminolevulinic acid (5-ALA) was used in all cases. The procedure was performed with intraoperative neuronavigation for planning and anatomical orientation. Depending on lesion location and functional risk, an awake-surgery protocol was adopted, and intraoperative neurophysiological monitoring was performed when indicated to maximise the extent of resection while preserving eloquent functions.

When required, diffusion-tensor-imaging (DTI) MRI with tractographic reconstruction of major eloquent subcortical pathways—including the corticospinal tract (CST), arcuate fasciculus (AF), optic radiation, and inferior fronto-occipital fasciculus (IFOF)—was acquired to optimise surgical planning and neuronavigation integration.

Starting in December 2023, pre-operative navigated transcranial magnetic stimulation (nTMS) was introduced for mapping cortical areas involved in language and motor function. These data were subsequently used to refine subcortical tractography, improving its accuracy and usefulness during planning and surgery.

From 2023 onward, intraoperative ultrasound was also implemented as an additional tool to support navigation and assess the extent of resection.

### 2.3. PET Analysis

Amino acid PET studies were acquired on two PET/CT systems: a Discovery MI Gen 2 (GE Medical Systems, LLC, Waukesha, WI, USA) and a Gemini TF PET/CT system (Philips Medical Systems, Cleveland, OH, USA). In accordance with the joint EANM/EANO/RANO practice guidelines, patients received a target intravenous injected activity of approximately 185 MBq of [^18^F]FET, subject to standard clinical tolerances. List-mode data were acquired. For standardised interpretation and semiquantitative analysis, a static image corresponding to the 20–40 min post-injection interval was reconstructed, as recommended to ensure comparability of FET-PET across protocols. For Scanner A (Philips Gemini TOF), images were reconstructed using a LOR-RAMLA algorithm with TOF, resulting in a matrix size of 128 × 128 and a voxel size of 2 × 2 × 2 mm. For Scanner B (GE Discovery MI Gen 2), a VUE Point FX reconstruction algorithm was applied (SharpIr, *Z*-axis Filter Standard, 3.5 mm Filter Cutoff, 34 subsets, 6 iterations), resulting in a matrix size of 256 x 256 and a voxel size of 1.17 × 1.17 × 1.8 mm. No ad-hoc harmonization procedures were performed. PET data were rigidly co-registered to recent MRI (3D post-contrast T1-weighted and 3D FLAIR, 1 mm isotropic voxels) prior to VOI definition.

Lesion analysis was performed on the fused 20–40 min image in close correlation with MRI to avoid nonspecific uptake, such as vascular structures. A contralateral crescent-shaped background VOI, encompassing both grey and white matter in the frontal region of the tumour-free hemisphere and extending over six adjacent transaxial slices was placed, following the standardised methodology for [^18^F]FET-PET [21,22]; its mean SUV (SUVbkg) served as the reference for all tumour-to-background ratios. A preliminary manual 3D VOI enclosing the lesion was drawn slice-by-slice to serve solely as the search region for automatic segmentation. Within this region, the metabolically active tumour volume was then delineated using a relative threshold of 1.6 × SUVbkg, consistent with PET RANO 1.0 [22]. All semiquantitative parameters (SUVmax, SUVmean, TBRmax, TBRmean and metabolic tumour volume (MTV) were derived from this threshold-defined volume. In FET-negative examinations, where no voxels reached the predefined ≥1.6 TBR threshold, automated segmentation could not be applied. In these cases, a manual 3D anatomical VOI was drawn to encompass the corresponding contrast-enhancing or T2/FLAIR hyperintense abnormality on the co-registered follow-up MRI. Semiquantitative parameters for these non-avid lesions, including TBRmean values mathematically lower than 1.6, were subsequently extracted from this MRI-guided anatomical VOI. The only exception was peak-uptake sampling: SUVpeak was computed as the mean SUV within a spherical VOI 1.4 cm in diameter centred on the hottest voxel. All VOIs and quantitative parameters were processed in LIFEx -v7.4.0.

For each scan, the following static parameters were evaluated:

- **SUVmax**—maximum tracer uptake within the lesion, reflecting the point of highest metabolic activity;
- **SUVpeak**—mean SUV within the 1.4 cm diameter spherical VOI centred on the hottest voxel, providing a more noise-robust estimate of peak uptake;
- **SUVmean**—mean tracer uptake within the threshold-defined metabolic tumour volume;
- **TBRmax**—ratio between SUVmax and mean background SUV;
- **TBRmean**—ratio between mean lesion uptake and mean background SUV, providing a global measure of metabolic activity;
- **TBRpeak**—ratio between SUVpeak and mean background SUV;
- **MTV**—volume of metabolically active tissue (TBR ≥ 1.6), reflecting the biological extent of disease.

### 2.4. MRI Analysis

MRI examinations were performed at our Institution using a 1.5-Tesla scanner until December 2023 (Ingenia, Philips, Best, The Netherlands) and 3-Tesla scanners from January 2024 to December 2025 (MAGNETOM Vida, Siemens Healthineers, Erlangen, Germany). The MRI protocol included standard morphological sequences: T1-weighted pre- and post-contrast, T2-weighted, FLAIR, and susceptibility-weighted imaging (SWI). Diffusion-weighted imaging (DWI) with ADC maps, perfusion-weighted imaging (PWI), and MR spectroscopy (MRS) were available only in a subset of examinations and were therefore not systematically included in the quantitative analysis.

### 2.5. Histopathological Analysis

Histomolecular analysis was performed on tumour samples obtained during surgical resection, following current diagnostic standards and integrating morphological evaluation, immunohistochemistry, and molecular testing when indicated. Histopathological characterisation included conventional histology with grading based on features consistent with high-grade glial neoplasms, such as hypercellularity, marked cytological atypia, variable tumour necrosis, and microvascular proliferation. Histopathological diagnosis was established according to the 2021 WHO Classification of Tumours of the Central Nervous System by integrating histomorphological findings with immunohistochemical and molecular analyses. Histological evaluation included assessment of tumour cellularity, nuclear atypia, mitotic activity, microvascular proliferation and necrosis.

Proliferative activity was assessed using the Ki-67 (MIB-1) index, and p53 expression was evaluated immunohistochemically. GFAP and OLIG2 immunostaining were also used to assess glial cell density and to support the identification of residual viable tumour cells in areas showing treatment-related changes.

IDH1/IDH2 mutational status was determined by immunohistochemistry (particularly IDH1 R132H) and/or molecular analysis via targeted Sanger sequencing or Next-Generation Sequencing (NGS), depending on availability and time period. Additional genomic alterations of prognostic and predictive relevance were investigated, including homozygous deletion of CDKN2A/B, EGFR amplification, TERT promoter status, and MGMT promoter methylation status assessed by methylation-specific PCR, according to laboratory protocols.

### 2.6. Data Evaluation

Histological, radiological, clinical, and PET assessments were performed by highly specialised personnel (neuropathologists, neuroradiologists, and nuclear medicine physicians) following standard procedures of our centre. Results were subsequently integrated and discussed in multidisciplinary neuro-meetings to define the final diagnostic interpretation and clinical-therapeutic management of each case.

The final diagnosis of tumour recurrence or TRC was established using a composite reference standard based on histopathological findings and clinical–radiological follow-up. Patients were classified as having tumour recurrence if one of the following criteria was met: (1) histopathological confirmation of tumour recurrence following re-surgery; (2) unequivocal progression on follow-up MRI performed 3 months after [^18^F]FET-PET in patients who did not undergo further surgery; or (3) clinical deterioration leading to best supportive care decision based on a markedly reduced Karnofsky Performance Status, followed by death attributable to tumour progression within 3–6 months after the PET examination.

A 3-month MRI follow-up interval was selected to preserve the temporal relationship between the PET findings and subsequent radiological evolution. Given the aggressive biology of these tumours, progression detected after a longer interval could not be reliably attributed to disease already present at the time of PET imaging. In other words, a longer interval (e.g., 6 months) could introduce bias in PET-negative cases, as the natural history of hHGGs entails a significantly increased probability of recurrence over such a timeframe, potentially misclassifying later events unrelated to the initial PET findings.

### 2.7. Statistical Analysis

All analyses were carried out in Python 3.14 (NumPy 2.4, SciPy 1.17, scikit-learn 1.8, pandas 3.0). Statistical significance was set at *p* < 0.05. Continuous variables were reported as median and interquartile range (IQR). Between-group differences (recurrence vs. TRC) were assessed with the Mann–Whitney U test, with the rank-biserial correlation as effect size; *p*-values were corrected for multiple comparisons using the Benjamini–Hochberg false discovery rate (FDR).

Diagnostic performance of each parameter was evaluated by receiver operating characteristic (ROC) analysis. Areas under the curve (AUC) were reported with 95% confidence intervals (DeLong method); optimal cut-offs were derived by the Youden index, with corresponding sensitivity and specificity. Internal validation used five-fold cross-validation stratified by outcome and grouped by patient, so that all examinations from the same patient were confined to a single fold and no information leaked between the training and test partitions. Within each training partition the Youden-optimal cut-off was re-derived de novo and then applied unchanged to the held-out partition; we reported the mean out-of-fold AUC with its between-fold standard deviation, the mean out-of-fold sensitivity and specificity, and the range of the fold-specific cut-offs. By applying this specific cross-validation, we expected an unbiased estimate of the performance from a Youden-derived threshold, without establishing the transportability of any individual numerical cut-off. Pairwise AUC comparisons were performed with the DeLong test for two pre-specified contrasts of clinical interest: TBRmax vs. TBRpeak and TBRmax vs. TBRmean.

The primary analysis included all 77 scans. As some patients contributed multiple scans, patient-level cluster bootstrap resampling was used to estimate the AUC confidence intervals. In addition, a sensitivity analysis restricted to one scan per patient (n = 61) was performed. Both analyses yielded results consistent with the primary analysis, indicating that the findings were not materially affected by within-patient clustering; the sensitivity analysis is reported in the Supplementary Material.

The diagnostic value of qualitative FET avidity was assessed by treating the presence or absence of pathological uptake as a binary classifier, computing sensitivity, specificity, positive and negative predictive values (95% Wilson CI), accuracy and AUC, with the association to outcome tested by Fisher’s exact test.

Given that scans were acquired on two different systems—an analogue scanner (Scanner A) and a digital SiPM-based scanner (Scanner B)—the effect of the acquisition system on quantification was assessed for both SUV and TBR metrics using the Mann–Whitney U test with Benjamini–Hochberg FDR correction. To verify that any between-scanner difference was not driven by an unequal distribution of recurrence and TRC across systems, each parameter was additionally compared with the van Elteren test (a stratified Wilcoxon rank-sum test, stratified by outcome); the outcome-adjusted *p*-value is reported.

## 3. Results

Between December 2019 and December 2025, 77 [^18^F]FET-PET procedures for a total of 61 patients with high-grade glioma were analysed. All of these patients underwent surgical resection and adjuvant chemoradiotherapy. The cohort included 38 men and 23 women, with a median age of 58 years (18–78). The initial tumour volume at diagnosis had a median of 27 cm^3^ (range 18–63 cm^3^), whereas at the time of suspected recurrence the median lesion volume was 1.9 cm^3^ (range 0.8–5.7 cm^3^). The interval between the first surgical procedure and the [^18^F]FET-PET performed for suspected recurrence had a median duration of 12.6 months (range 3.2–102.9 months).

The histological distribution at initial surgery, classified according to WHO CNS criteria, is reported in Table 1 and consisted of IDH-wildtype glioblastoma (grade 4) in 73,8% of cases, IDH-mutant astrocytoma grades 3 in 11,5%, astrocytoma grade 4 in 1,6% and IDH-mutant oligodendroglioma with 1p/19q codeletion (grade 3) in 13,1%. All patients underwent GTR, with systematic use of 5-ALA and intraoperative neuronavigation. All reoperated patients underwent histopathological assessment, which confirmed tumour recurrence in every case; no reoperated case was classified as TRC. Depending on lesion location and functional risk, intraoperative neurophysiological monitoring was used in 24 cases, awake surgery in 5 cases, DTI-tractography in 40 cases, pre-operative nTMS in 13 cases, and intraoperative ultrasound in 16 cases. Clinical and demographic characteristics are summarised in Table 1.

Among FET-positive examinations (n = 65), the final diagnosis was verified by histopathology in 29, 3-month radiological follow-up in 30, and clinical deterioration/death attributable to progression in 6; among FET-negative examinations (n = 12), verification was based on 3-month radiological follow-up.

**Figure 1:**
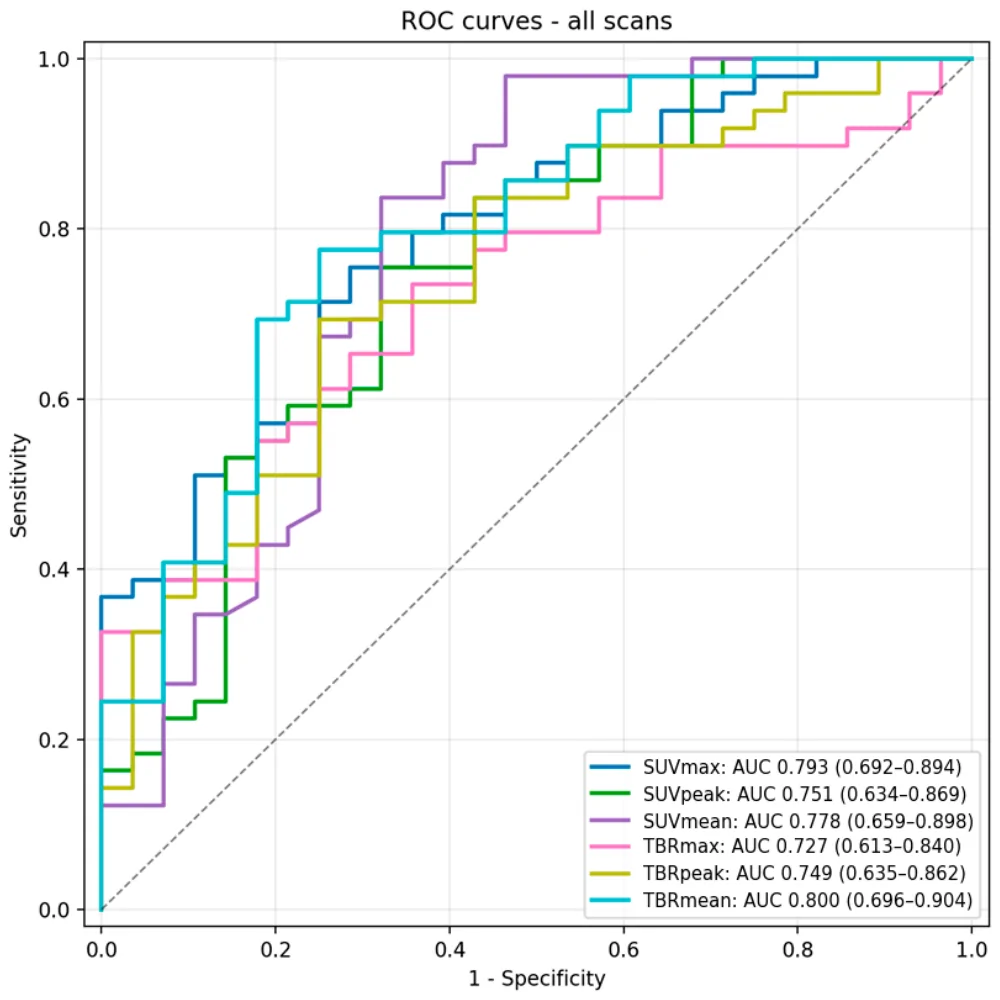
Receiver operating characteristic (ROC) curves of static [^18^F]FET-PET parameters for distinguishing tumour recurrence from treatment-related changes (TRC) in all scans (n = 77). AUCs with 95% confidence intervals are shown in the legend; the dashed line indicates chance-level performance.

All semiquantitative parameters except metabolic tumour volume (MTV) were significantly higher in the recurrence group than in the TRC group (Mann–Whitney U test, FDR-corrected; Table 2, Figure 1), with moderate-to-large effect sizes (rank-biserial r 0.45–0.60).

On ROC analysis (Table 3), TBRmean showed the highest discriminative accuracy (AUC 0.800, 95% CI 0.696–0.904), followed by SUVmax (AUC 0.793, 95% CI 0.692–0.894) and SUVmean (AUC 0.778, 95% CI 0.659–0.898). TBRmax yielded an AUC of 0.727 (95% CI 0.613–0.840). Cross-validated AUCs were consistent with the apparent values (e.g., TBRmean 0.815 vs. 0.800; SUVmax 0.795 vs. 0.793), indicating limited optimism bias, although fold-to-fold variability was non-negligible for some parameters (SD up to 0.15) given the cohort size. Because the Youden cut-off was re-derived within each training partition and applied to the held-out partition, the cross-validated sensitivity and specificity reported in Table 3 further quantified the optimism of the thresholds themselves. Optimism was modest for TBRmean (sensitivity 77.6% to 71.3%; specificity 75.0% to 68.7%) and SUVmax (75.5% to 73.8%; 71.4% to 64.0%), and the corresponding fold-specific cut-offs varied within a narrow range (TBRmean 1.83–1.91; SUVmax 2.37–2.57). By contrast, TBRmax showed a substantial loss of out-of-fold sensitivity (73.5% to 57.1%) together with a wider spread of fold-specific cut-offs (2.69–3.18), and the MTV threshold was frankly unstable (0.72–21.90), consistent with its non-discriminative AUC.

In the two pre-specified DeLong comparisons, no significant difference in AUC was found between TBRmax and TBRpeak (ΔAUC −0.022; *p* = 0.485) or between TBRmax and TBRmean (ΔAUC −0.074; *p* = 0.133; Table 4).

The presence of pathological FET uptake identified all recurrences (49/49; sensitivity 100%, 95% CI 92.7–100), with a negative predictive value of 100% (95% CI 75.8–100), specificity of 42.9% (95% CI 26.5–60.9), positive predictive value of 75.4% and overall accuracy of 79.2% (Fisher’s exact test, *p* < 0.001; Table 5). When restricted to FET-avid lesions (n = 65), MTV showed no association with outcome, either as a group difference (median 7.5 vs. 6.6 cm^3^; *p* = 0.994) or as a continuous classifier (AUC 0.499). Thus, while the presence of FET uptake was highly informative, the magnitude of the metabolically active volume was not.

**Figure 2:**
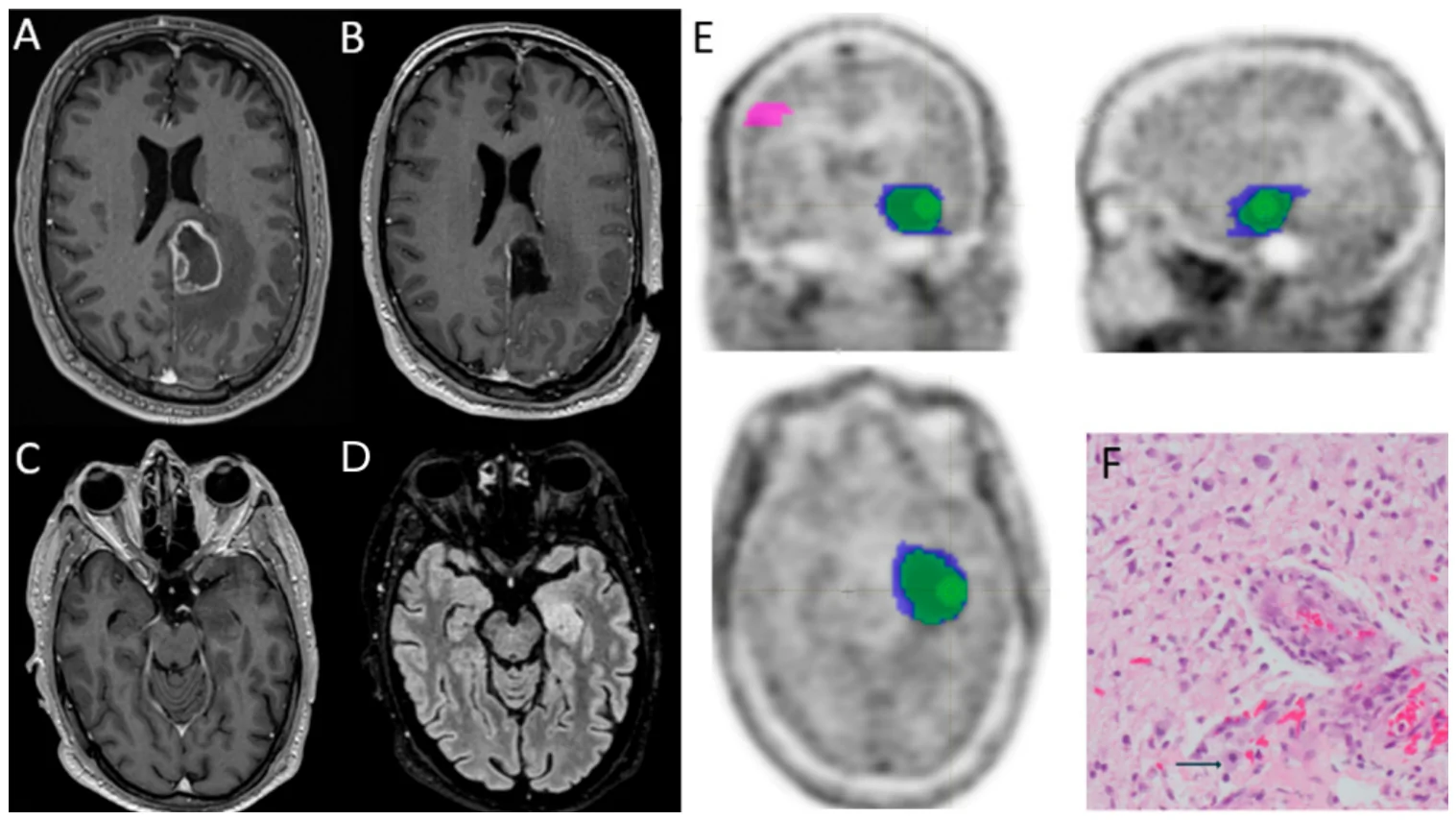
(**A**) Preoperative gadolinium-enhanced MRI demonstrating a high-grade left parietomesial glioma. (**B**) Early postoperative MRI showing no residual pathological contrast enhancement. (**C**) Follow-up MRI performed 1 year later, showing only faint contrast enhancement. (**D**) a Follow-up MRI performed 1 year later: clearly hyperintense lesion on T2-weighted/FLAIR sequences in the left mesial temporal region, raising suspicion of tumour progression. (**E**) Subsequent [^18^F]FET-PET demonstrating increased tracer uptake in the left mesial temporal region (SUVmax, 4.43; SUVpeak, 3.15; SUVmean, 2.19; TBRmax, 4.99; TBRpeak, 3.55; TBRmean, 2.47), consistent with metabolically active tumour. (**F**) Histopathological confirmation of tumour recurrence. High-power hematoxylin and eosin staining (40× magnification) shows a diffusely infiltrating high-grade glial neoplasm characterised by marked hypercellularity, prominent microvascular proliferation, and pleomorphic tumour cells within a fibrillary background. Neoplastic astrocytic cells exhibit pronounced cytological atypia, including enlarged hyperchromatic nuclei, marked nuclear pleomorphism, and mitotic figures (black arrow). VOI colour coding: pink, background VOI; blue, manual 3D VOI (search region); green, threshold-defined metabolic tumour volume (1.6 × SUV_bkg). The 1.4 cm diameter sphere used for SUV_peak sampling is also visible.

**Figure 3:**
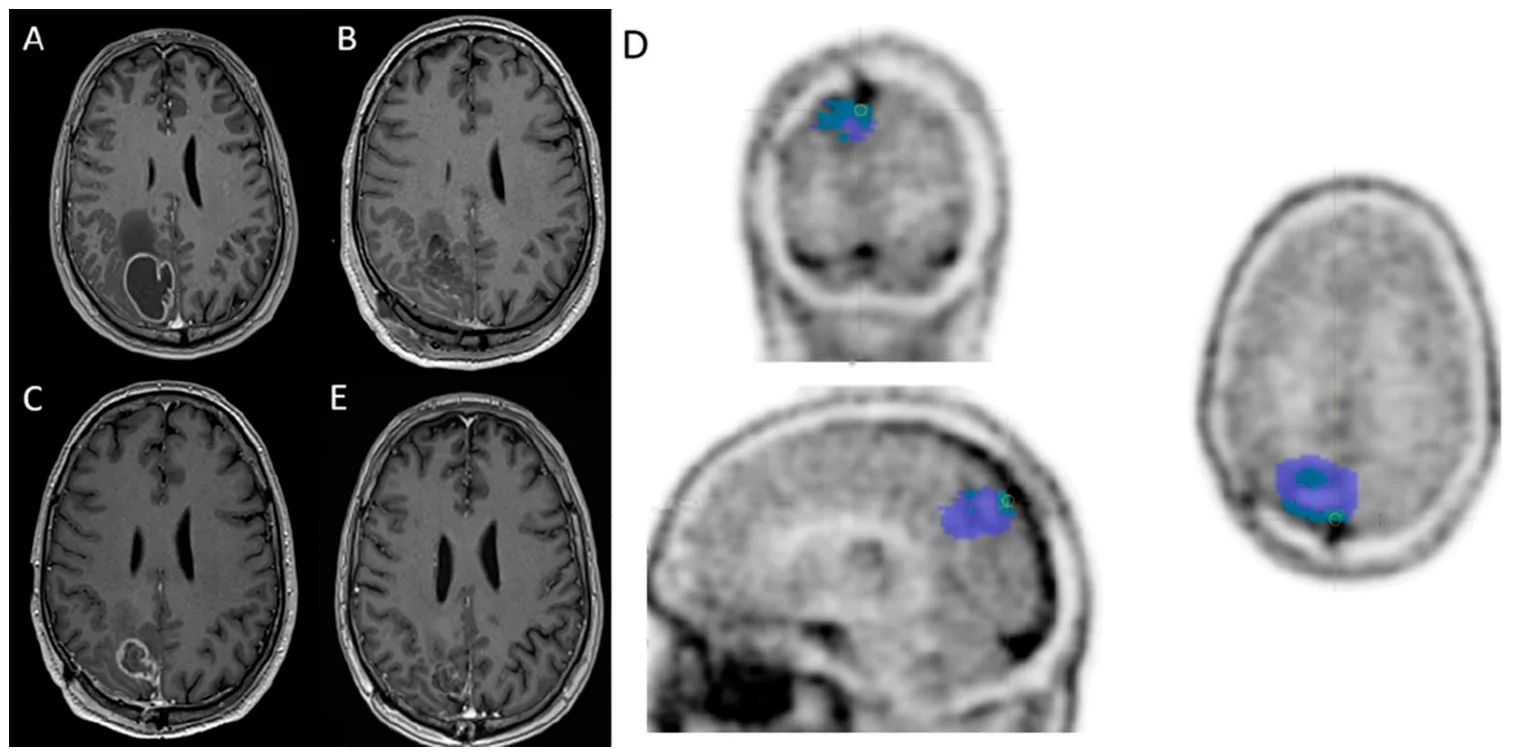
(**A**) Preoperative gadolinium-enhanced MRI demonstrating a high-grade glioma involving the right parieto-occipital region. (**B**) Early postoperative MRI demonstrating complete resection of the contrast-enhancing tumour. (**C**) Follow-up contrast-enhanced T1-weighted MRI performed 9 months after completion of adjuvant chemoradiotherapy, showing new enhancement along the margin of the resection cavity and raising suspicion of tumour recurrence. (**D**) Subsequent [^18^F]FET-PET/CT showing only mildly increased tracer uptake, with all semiquantitative parameters falling below the cut-offs derived in this cohort (SUVmax, 1.82 [cut-off 2.53]; SUVpeak, 1.38 [1.88]; SUVmean, 1.26 [1.61]; TBRmax, 2.61 [2.69]; TBRpeak, 1.97 [2.04]; TBRmean, 1.81 [1.85]), arguing against tumour recurrence (**E**) The patient underwent close radiological follow-up. MRI performed 3 months later showed near-complete resolution of the contrast enhancement, supporting a diagnosis of treatment-related changes rather than tumour recurrence. VOI colour coding: blue, manual 3D VOI (search region); green, threshold-defined metabolic tumour volume (1.6 × SUV_bkg).

Representative imaging examples are shown in Figure 2 and Figure 3. Figure 2 illustrates a FET-avid lesion subsequently confirmed as tumour recurrence by histopathological findings, whereas Figure 3 shows a lesion with mildly increased uptake and semiquantitative values below the cohort-derived thresholds, subsequently classified as treatment-related change based on radiological follow-up.

The distribution of recurrence and TRC was comparable across the two systems (recurrence 62.5% on Scanner A vs. 66.7% on Scanner B), limiting the potential for case-mix confounding. Background uptake did not differ between the analogue (Scanner A, n = 56) and digital SiPM-based (Scanner B, n = 21) systems (SUVbkg 0.92 vs. 0.89; *p* = 0.66). Among the tumour metrics, between-system differences in both SUVmax and TBRmax remained significant after adjustment for outcome (adjusted *p* = 0.006 and *p* = 0.003, respectively). By contrast, no significant between-system differences were observed for SUVmean (*p* = 0.378), TBRpeak (*p* = 0.148), or, importantly, TBRmean (unadjusted *p* = 0.564; outcome-adjusted *p* = 0.333). Thus, background normalisation alone did not confer robustness to the acquisition platform: maximum-based indices remained scanner-dependent whether or not they were normalised to background, whereas TBRmean was the only tumour-to-background metric combining high discrimination with no significant between-scanner difference in this cohort (Table 6). Representative clinical cases illustrating the qualitative and semiquantitative evaluation of [^18^F]FET-PET findings for the differentiation between tumour recurrence and TRC are provided in Figure 2 and Figure 3.

### Supplementary Material—Per-Patient Sensitivity Analysis

To assess the influence of including multiple scans from the same patient, the entire inferential analysis was repeated retaining one scan per patient (n = 61; n = 61; when multiple scans were available, the positive scan was retained). Results were consistent with the primary all-scans analysis in the direction and magnitude of effects, supporting the robustness of the main findings to the unit of analysis (Tables S1–S4).

## 4. Discussion

In this study, we systematically compared the diagnostic performance of static [^18^F]FET-PET semiquantitative parameters for differentiating tumour recurrence from TRC in previously treated HGGs. All parameters except MTV showed moderate-to-good discriminative accuracy, with AUCs ranging from 0.73 to 0.80, significant between-group differences after correction for multiple comparisons, and moderate-to-large effect sizes. MTV was therefore the only parameter that did not show comparable discriminatory performance in this cohort. From a biological perspective, this suggests that treatment-related changes can manifest as diffuse areas of tissue injury with moderately increased amino acid transport over large volumes, resulting in MTV values that overlap significantly with true tumour recurrences. Conversely, uptake intensity metrics (SUV and TBR) act as better proxies for the dense cellularity and high metabolic demand characteristic of active tumour cores. Furthermore, this aligns with the current joint EANM/EANO/RANO practice guidelines, which provide validated thresholds for TBR parameters to distinguish recurrence from treatment-related changes, but do not endorse any volumetric MTV cut-off for this specific differential diagnosis [8]. Overall, these findings support the established role of amino-acid PET as a complementary modality when conventional MRI is inconclusive, owing to its ability to depict increased amino-acid transport with limited dependence on blood–brain barrier disruption [5,8,9].

Notably, the qualitative absence of focal [^18^F]FET uptake showed 100% sensitivity and a 100% negative predictive value, although specificity was limited to 43%. Within this cohort, the absence of focal uptake therefore represented a strong rule-out finding, whereas the presence of focal uptake alone was insufficient to establish tumour recurrence. Clinically, qualitative assessment may thus serve as an initial rule-out step: the absence of focal uptake may identify lesions unlikely to represent recurrence, whereas uptake-positive lesions require semiquantitative assessment to improve differentiation between recurrence and TRC (Figure 2 and Figure 3).

TBRmean and SUVmax showed the highest diagnostic accuracy and the strongest cross-validated performance (Table 3). However, the pre-specified pairwise comparisons between TBR-derived parameters—TBRmax versus TBRpeak and TBRmax versus TBRmean—did not reach statistical significance (Table 4). The present study therefore does not establish the superiority of any single static parameter. Instead, the best-performing indices should be regarded as comparably informative measures that capture partly different aspects of lesion uptake. SUVmax remains a clinically attractive option because it is rapidly obtained and does not require averaging across the lesion volume. Nevertheless, absolute SUVmax values differed significantly between the two PET systems used in this study (Table 6), cautioning against transferring the internally derived cutoff of approximately 2.53 across institutions without technical harmonisation and external validation. Notably, this scanner dependence was not confined to absolute SUV: TBRmax was equally affected, indicating that background normalisation does not by itself neutralise platform-related variability when the numerator is derived from a single hottest voxel. A likely contributor is the different spatial resolution of the two systems: the digital SiPM-based scanner provides higher spatial resolution and improved time-of-flight performance than the analogue PMT-based system, resulting in a smaller partial-volume effect and, consequently, greater recovery of maximum activity. Because maximum-based indices are sampled from the single hottest voxel, they are the most susceptible to partial-volume averaging and image noise, so the higher SUVmax and TBRmax observed on the digital scanner reflect differences in resolution recovery rather than a genuinely higher tracer uptake. This effect is particularly relevant in our cohort, where recurrent lesions were typically small (median 1.9 cm^3^) and therefore especially prone to partial-volume-related underestimation on the lower-resolution system. These findings reinforce the need to interpret quantitative thresholds within locally validated imaging pipelines.

Although TBRmean was not statistically superior to the other TBR-based indices, it may represent the most suitable operational primary quantitative marker in this cohort. Unlike the maximum-based metrics, TBRmean did not differ significantly between the two PET systems. This indicates that stability across the acquisition platform is conferred not by background normalisation alone (which TBRmax also applies) but by averaging uptake over the metabolically active volume, which is inherently less sensitive to voxel-level noise and to the partial-volume effects that differ between scanners of unequal spatial resolution. This scanner robustness, together with the highest observed AUC and the superior specificity relative to TBRmax, supports the pragmatic selection of TBRmean as the primary quantitative marker. Its use is also consistent with current EANM/EANO/RANO recommendations favouring tumour-to-background ratios for amino-acid PET assessment [8]. This preference should therefore be interpreted as pragmatic and methodologically motivated rather than as evidence of greater intrinsic diagnostic accuracy.

Comparison with previous studies supports the diagnostic relevance of TBRmean while also illustrating the heterogeneity of the available evidence. Direct numerical comparison is complicated by differences in the static parameters evaluated (TBRmean vs. TBRmax), analytical methods, acquisition and reconstruction protocols, and proposed thresholds. For example, Celli et al. reported 86% sensitivity and 81% specificity for TBRmax at a cutoff of 2.1, whereas other single-centre investigations used different analytical approaches and thresholds [19,23]. More specifically for TBRmean, Werner et al. reported a cutoff of 1.95, yielding an AUC of 0.89 and an overall accuracy of 83% [24]. Likewise, in a larger cohort of 151 patients, Müller et al. found significantly higher TBRmean values in tumour progression than in TRC (2.1 ± 0.3 vs. 1.9 ± 0.3; *p* < 0.001). In that study, a logistic regression model combining TBRmean and TBRmax achieved an AUC of 0.78, with 64% sensitivity and 80% specificity in the validation cohort [25]. In our cohort, the optimal TBRmean cutoff of approximately 1.85 yielded 78% sensitivity, 75% specificity, and an overall accuracy of 77%. This value is broadly consistent with previous TBRmean findings, although slightly lower than the TBRmean threshold of 2.0 traditionally cited in broader guideline summaries [8].

These comparisons should not, however, be interpreted as evidence that TBR thresholds are universally transferable. TBR values are not intrinsic biological constants and may be influenced by patient population, acquisition and reconstruction protocols, post-processing procedures, lesion and background VOI definition, segmentation strategy, and voxel-inclusion criteria [5,25,26,27,28]. TBRmean may be particularly sensitive to the delineation of heterogeneous lesions and to the inclusion of necrotic, postoperative, or otherwise non-representative tissue, whereas maximum-based indices are more susceptible to voxel-level noise and reconstruction settings [26,27]. Thus, although normalisation to reference tissue may make TBRmean less sensitive than absolute SUV measures to between-scanner shifts, it does not make it fully independent of the quantitative imaging pipeline.

Regarding TBRmax, the reported thresholds in previous cohorts generally fall within a relatively restricted range. Individual studies have frequently identified optimal TBRmax cutoffs between 1.95 and 2.10 [19,29,30], while meta-analytic evidence has reported a broader range of approximately 1.9–2.3 [31]. Although higher than most previously reported values, the locally derived TBRmax cutoff of 2.69 remained within the broader spectrum of methodological variability observed across studies, reinforcing the view that published thresholds should be considered indicative ranges requiring local validation. The fact that our optimal TBRmean threshold (1.85) is lower than our TBRmax threshold (2.69) is mathematically expected, as TBRmean averages uptake across the entire active metabolic volume rather than isolating the single highest-uptake voxel. Nevertheless, minor threshold differences among studies remain clinically relevant and reflect variations in parameter selection, patient case mix, scanner characteristics, image reconstruction, post-processing, and VOI methodology. Accordingly, published values should be regarded as a reference range rather than as universally applicable decision thresholds.

The static semiquantitative uptake parameters evaluated in our study demonstrated a robust and narrowly distributed diagnostic performance, with AUCs ranging from 0.73 to 0.80. This remarkable consistency across analytically distinct metrics highlights the inherent clinical robustness of [^18^F]FET-PET for differentiating true tumour recurrence from TRC in HGGs. This comparability has important practical implications, suggesting that the main challenge in the clinical implementation of static [^18^F]FET-PET is not the identification of a universally optimal semiquantitative parameter, but the establishment of reproducible and technically consistent quantitative workflows. Institutions should therefore prioritise standardised approaches that are compatible with their scanner infrastructure and post-processing pipelines and that enable reliable interpretation within the local imaging environment. Centre-specific calibration may represent a pragmatic implementation step, particularly when more than one PET system is used. Nevertheless, locally derived cutoffs should be pre-specified, prospectively monitored, and externally validated where possible. Local optimisation should therefore complement, rather than replace, broader efforts to harmonise acquisition, reconstruction, segmentation, and quantitative reporting across centres. Consistent with this interpretation, previous studies have suggested that combining static and dynamic PET parameters with MRI findings may further improve the diagnostic assessment of suspected tumour recurrence [32,33,34,35,36].

Several limitations should be considered. Most importantly, the optimal decision thresholds identified in this cohort were not prospectively tested in an independent external validation cohort. Internal cross-validation supports the stability of the estimates within the available dataset, but it does not establish their diagnostic performance in populations evaluated with different scanners, acquisition protocols, or quantitative pipelines. Internal validation was nevertheless applied to the thresholds themselves and not only to the AUC: cut-offs were re-derived within each training partition and evaluated out of fold, and the resulting estimates showed limited optimism for TBRmean and SUVmax but appreciable instability for TBRmax. Cross-validation of this kind estimates the performance to be expected from a Youden-derived threshold within this cohort; it does not establish the transportability of the specific numerical values. The proposed cut-offs should therefore be regarded as cohort-derived thresholds with internally cross-validated performance estimates, requiring prospective external validation, rather than as universally applicable decision thresholds. However, this limitation further underlines the practical value of our approach. Given that mathematical cutoffs are intrinsically adapted to the single-centre imaging environment, the adoption of locally optimised thresholds represents the most effective strategy to safeguard diagnostic adequacy in daily clinical practice. Additionally, the lack of direct, spatially matched correlation between PET volumetric parameters, quantitative MRI volumes, and specific histopathological markers (such as cellular density or macrophage infiltration) limits our ability to provide a definitive mechanistic explanation for the non-discriminative nature of MTV. Future prospective studies incorporating precise radiopathologic co-registration are still needed to fully elucidate this aspect. Furthermore, the inclusion of different HGG histomolecular entities introduces biological and clinical heterogeneity, which may influence tracer uptake, patterns of recurrence, and disease course. In addition, the comparison between PET systems was unpaired. Therefore, the absence of a significant between-scanner difference for TBRmean cannot establish reproducibility or scanner equivalence, and residual confounding related to patient, tumour, treatment, or technical characteristics cannot be excluded. Finally, the reference standard combined histopathology with imaging and clinical follow-up. Although this approach reflects routine clinical practice, it introduces heterogeneity in outcome ascertainment (histopathological versus clinico-radiological) and the possibility of verification bias or misclassification. Because histological verification was not uniformly available and subsequent management may have been influenced by FET-PET findings, differential verification bias cannot be excluded and may have contributed to the observed 100% sensitivity and negative predictive value of qualitative FET uptake.

## 5. Conclusions

Static semiquantitative [^18^F]FET-PET parameters reliably differentiated tumour recurrence from TRC in HGGs. The relatively consistent diagnostic performance observed across PET measurements further supports the overall robustness and reliability of [^18^F]FET-PET in this setting. Diagnostic accuracy was established against a composite reference standard integrating histopathology with longitudinal clinical and radiological follow-up, demonstrating that PET findings align directly with true clinical outcomes. Among these parameters, TBRmean achieved the highest diagnostic accuracy and was favoured as the primary quantitative marker because it combined strong diagnostic discrimination and specificity with cross-scanner stability. However, it should be acknowledged that no static parameter demonstrated statistically superior diagnostic performance. Although the optimal values identified in our cohort differed slightly from those reported in current guidelines, such discrepancies are expected given differences in patient populations and technical methodology. Rather than pursuing a universally applicable cutoff, guideline-recommended thresholds should therefore be regarded as a reference framework to be locally validated and, when necessary, calibrated according to centre- and protocol-specific conditions. Ultimately, these quantitative parameters should not be interpreted in isolation, but rather integrated within the broader clinical, radiological, and multidisciplinary decision-making framework.

## Figures and Tables

**Table 1:** Baseline demographic, histological, and surgical characteristics of the study cohort.

Characteristic	Value
Number of patients	**61**
Number of FET-PET examinations	**77**
Male sex, n (%)	**38 (62.3%)**
Female sex, n (%)	**23 (37.7%)**
Median age, years	**58 (18–78)**
Interval first surgery–FET-PET, median (range), months	**12.6 (3.2–102.9)**
Glioblastoma, IDH-wildtype (WHO grade 4)	**45 (73.8%)**
Astrocytoma, IDH-mutant (WHO grade 3)	**7 (11,5%)**
Astrocytoma, IDH-mutant (WHO grade 4)	**1 (1,6%)**
Oligodendroglioma, IDH-mutant, 1p/19q-codeleted (WHO grade 3)	**8 (13.1%)**
Neurophysiological monitoring, n (%)	**24 (39.3%)**
Awake surgery, n (%)	**5 (8.2%)**
Diffusion tensor imaging (DTI), n (%)	**40 (65.6%)**
Pre-operative navigated TMS, n (%)	**13 (21.3%)**
Intraoperative ultrasound, n (%)	**16 (26.2%)**

**Table 2:** Descriptive statistics and group comparison (all scans, n = 77).

Parameter	Recurrence Median (IQR)	TRC Median (IQR)	*p* (FDR)	r
SUVmax	3.11 (2.53–4.20)	2.18 (1.87–2.63)	<0.001	0.59
SUVpeak	2.19 (1.88–2.65)	1.71 (1.37–2.04)	<0.001	0.50
SUVmean	1.89 (1.67–2.23)	1.30 (0.89–1.81)	<0.001	0.56
TBRmax	3.25 (2.60–4.38)	2.42 (2.06–3.01)	0.001	0.45
TBRpeak	2.24 (1.94–2.79)	1.89 (1.70–2.07)	<0.001	0.50
TBRmean	2.01 (1.88–2.21)	1.75 (1.08–1.86)	<0.001	0.60
MTV (cm^3^)	7.54 (2.37–16.47)	6.62 (2.76–15.20)	0.994	−0.003

Recurrence, n = 49; TRC, n = 28. Median (IQR) values; *p* from Mann–Whitney U test with Benjamini–Hochberg FDR correction; effect size as rank-biserial correlation (r). SUVmax, maximum uptake within the threshold-defined tumour volume; SUVpeak, mean SUV within a 1.4 cm-diameter sphere on the hottest voxel; SUVmean, mean uptake within the threshold-defined volume; TBRmax/TBRmean/TBRpeak, corresponding tumour-to-background ratios (denominator: mean background SUV); MTV, metabolically active tumour volume (threshold 1.6 × background), computed only in FET-avid lesions (recurrence n = 49, TRC n = 16). TRC, treatment-related changes.

**Table 3:** ROC analysis of static parameters (all scans, n = 77).

Parameter	Cut-Off	Sens. Apparent → CV (%)	Spec. Apparent → CV (%)	Fold-Specific Cut-Offs
*SUVmax*	*2.53*	*75.5 → 73.8*	*71.4 → 64.0*	*2.37–2.57*
*SUVpeak*	*1.88*	*75.5 → 77.8*	*67.9 → 56.0*	*1.75–1.88*
*SUVmean*	*1.61*	*83.7 → 85.8*	*67.9 → 58.0*	*1.32–1.65*
*TBRmax*	*2.69*	*73.5 → 57.1*	*64.3 → 64.0*	*2.69–3.18*
*TBRpeak*	*2.04*	*69.4 → 68.9*	*75.0 → 68.0*	*1.90–2.05*
*TBRmean*	*1.85*	*77.6 → 71.3*	*75.0 → 68.7*	*1.83–1.91*
*MTV*	*0.72*	*89.8 → 64.2*	*18.8 → 23.3*	*0.72–21.90*

AUC with 95% CI (DeLong method), Youden-optimal cut-off, sensitivity and specificity. Sens-CV and Spec-CV, mean out-of-fold sensitivity and specificity obtained by re-deriving the Youden cut-off within each training partition and applying it unchanged to the held-out partition; the Cut-off, Sens. and Spec. columns are apparent (whole-cohort) values. n = 77 for all parameters except MTV (n = 65 FET-avid lesions). Patient-level cluster-bootstrap 95% CIs (10,000 resamples) were near-identical to the DeLong intervals for all parameters (e.g., TBRmean 0.687–0.903 vs. 0.696–0.904; SUVmax 0.683–0.892 vs. 0.692–0.894), confirming a negligible effect of within-patient clustering.

**Table 4:** Pairwise AUC comparisons (DeLong test).

Comparison	AUC 1	AUC 2	ΔAUC	*p*
TBRmax vs. TBRpeak	0.727	0.749	−0.022	0.485
TBRmax vs. TBRmean	0.727	0.800	−0.074	0.133

Comparison of correlated ROC curves (DeLong test) for the two pre-specified contrasts. Neither reached statistical significance, indicating comparable performance among tumour-to-background metrics.

**Table 5:** Diagnostic performance of qualitative FET uptake (all scans, n = 77).

Metric	Value (95% CI)
True positives/False positives	49/16
False negatives/True negatives	0/12
Sensitivity	100% (92.7–100)
Specificity	42.9% (26.5–60.9)
Positive predictive value	75.4% (63.7–84.2)
Negative predictive value	100% (75.8–100)
Accuracy	79.2%
AUC	0.714 (0.621–0.808)
Fisher’s exact test	*p* < 0.001

Presence versus absence of pathological [^18^F]FET uptake as a binary classifier of recurrence/progression. 95% confidence intervals by the Wilson method. Sensitivity and negative predictive value are both 100% because no false-negative case occurred; this reflects, in part, the differential verification of positive and negative cases (verification bias) and should be interpreted accordingly.

**Table 6:** Scanner effect on SUV and TBR metrics.

Parameter	Scanner A (n = 56)	Scanner B (n = 21)	*p* (M–W)	q (FDR)	*p* (Adj. Outcome)
SUVbkg	0.92	0.89	0.660	0.660	0.618
SUVmax	2.53	3.39	0.008	0.035	0.006
SUVpeak	1.89	2.23	0.060	0.140	0.051
SUVmean	1.75	1.89	0.378	0.530	0.569
TBRmax	2.71	3.50	0.010	0.035	0.003
TBRpeak	2.03	2.23	0.148	0.259	0.089
TBRmean	1.90	1.94	0.564	0.657	0.333

Median values per system; *p* from the Mann–Whitney U test with Benjamini–Hochberg FDR correction (q); outcome-adjusted *p* from the van Elteren test (stratified Wilcoxon rank-sum test, stratified by outcome). Maximum-based indices (SUVmax and TBRmax) were significantly higher on the digital scanner and remained so after outcome adjustment, whereas background uptake, SUVmean, TBRpeak and TBRmean were unaffected.

## Data Availability

The data supporting the findings of this study are not publicly available. Data sharing is restricted by patient confidentiality requirements, applicable data-protection regulations, and the conditions of the ethical approval.

## Supplementary Materials

The following supporting information can be downloaded at: https://www.mdpi.com/article/10.3390/brainsci16101085/s1, Table S1: Descriptive statistics and group comparison (one scan per patient, n = 61); Table S2: ROC analysis (one scan per patient, n = 61); Table S3: Pairwise AUC comparisons—DeLong test (one scan per patient, n = 61); Table S4: Diagnostic performance of qualitative FET uptake (one scan per patient, n = 61).

## Abbreviations

5-ALA	5-aminolevulinic acid
ADC	apparent diffusion coefficient
AF	arcuate fasciculus
AUC	area under the receiver operating characteristic curve
CDKN2A/B	cyclin-dependent kinase inhibitor 2A/2B
CI	confidence interval
CNS	central nervous system
CST	corticospinal tract
CT	computed tomography
DTI	diffusion tensor imaging
DWI	diffusion-weighted imaging
EANM	European Association of Nuclear Medicine
EANO	European Association of Neuro-Oncology
EGFR	epidermal growth factor receptor
[^18^F]FET	O-(2-[^18^F]fluoroethyl)-L-tyrosine
FDR	false discovery rate
FLAIR	fluid-attenuated inversion recovery
GTR	gross total resection
HGG	high-grade glioma
IDH	isocitrate dehydrogenase
IFOF	inferior fronto-occipital fasciculus
IQR	interquartile range
MGMT	O^6^-methylguanine-DNA methyltransferase
MRI	magnetic resonance imaging
MRS	magnetic resonance spectroscopy
MTV	metabolic tumour volume
NGS	next-generation sequencing
NPV	negative predictive value
nTMS	navigated transcranial magnetic stimulation
PET	positron emission tomography
PWI	perfusion-weighted imaging
RANO	Response Assessment in Neuro-Oncology
ROC	receiver operating characteristic
SD	standard deviation
SiPM	silicon photomultiplier
SUV	standardised uptake value
SUVbkg	mean background standardised uptake value
SUVmax	maximum standardised uptake value
SUVmean	mean standardised uptake value
SUVpeak	peak standardised uptake value
SWI	susceptibility-weighted imaging
TBR	tumour-to-background ratio
TBRmax	maximum tumour-to-background ratio
TBRmean	mean tumour-to-background ratio
TBRpeak	peak tumour-to-background ratio
TERT	telomerase reverse transcriptase
TOF	time of flight
TRC	treatment-related changes
TTP	time to peak
VOI	volume of interest
