# Supplementary material for: Beyond a Universal Cut-Off: [18F]FET-PET Metrics for Distinguishing High-Grade Glioma Recurrence from Treatment-Related Changes
Source: Brain Sci. 2026 Oct 9;16(10):1085. doi: 10.3390/brainsci16101085 (PMC13702475; doi:10.3390/brainsci16101085)
Supplement: Supplementary file 1 [file brainsci-16-01085-s001.zip › brainsci-4508342-supplementary.pdf]

Supplementary Table S1. Descriptive statistics and group comparison (one scan per patient, n = 61).

Parameter	Recurrence median (IQR)	TRC median (IQR)	p (FDR)	r
SUVmax	3.27 (2.57–4.36)	2.33 (1.90–2.73)	<0.001	0.62
SUVpeak	2.31 (1.90–2.72)	1.74 (1.43–2.07)	0.003	0.49
SUVmean	1.94 (1.70–2.30)	1.47 (1.00–1.82)	<0.001	0.56
TBRmax	3.27 (2.62–4.38)	2.45 (2.05–3.09)	0.005	0.45
TBRpeak	2.22 (1.94–2.79)	1.96 (1.69–2.22)	0.007	0.44
TBRmean	1.99 (1.88–2.20)	1.75 (1.13–1.89)	<0.001	0.57
MTV (cm^3)	8.27 (2.47–17.23)	8.84 (2.76–18.74)	0.860	−0.04

Supplementary Table S1. Sensitivity analysis restricted to one scan per patient (recurrence n = 42, TRC n = 19). Median (IQR) values; p from the Mann–Whitney U test with Benjamini–Hochberg FDR correction; effect size as rank-biserial correlation (r). Parameter definitions as in Table 2 of the main text. MTV was computed only in FET-avid lesions (recurrence n = 42, TRC n = 12).

Supplementary Table S2. ROC analysis (one scan per patient, n = 61).

Parameter	AUC (95% CI)	Cut-off	Sens. (%)	Spec. (%)	AUC-CV (SD)
SUVmax	0.810 (0.695–0.924)	2.57	76.2	73.7	0.807 (0.09)
SUVpeak	0.743 (0.597–0.889)	1.88	81.0	68.4	0.722 (0.14)
SUVmean	0.778 (0.634–0.921)	1.61	85.7	68.4	0.753 (0.13)
TBRmax	0.724 (0.593–0.856)	2.57	78.6	57.9	0.716 (0.07)
TBRpeak	0.719 (0.575–0.863)	1.84	92.9	47.4	0.720 (0.07)
TBRmean	0.786 (0.660–0.911)	1.91	69.0	78.9	0.795 (0.11)
MTV	0.482 (0.287–0.677)	2.13	83.3	25.0	0.526 (0.21)

Supplementary Table S2. AUC with 95% CI (DeLong), Youden-optimal cut-off, sensitivity and specificity. AUC-CV: mean AUC from stratified five-fold cross-validation (SD in parentheses). n = 61, except MTV (n = 54 FET-avid lesions). SUVmax was the top performer in this analysis (AUC 0.810), with TBRmean closely comparable (AUC 0.786), consistent with the main analysis.

Supplementary Table S3. Pairwise AUC comparisons — DeLong test (one scan per patient, n = 61).

Comparison	AUC 1	AUC 2	ΔAUC	p
TBRmax vs TBRpeak	0.724	0.719	0.005	0.904
TBRmax vs TBRmean	0.724	0.786	−0.061	0.201

Supplementary Table S3. Pre-specified pairwise comparisons of correlated ROC curves (DeLong test). As in the main analysis, no comparison reached statistical significance.

Supplementary Table S4. Diagnostic performance of qualitative FET uptake (one scan per patient, n = 61).

Metric	Value (95% CI)
True positives / False positives	42 / 12
False negatives / True negatives	0 / 7
Sensitivity	100% (91.6–100)
Specificity	36.8% (19.1–59.0)
Positive predictive value	77.8% (65.1–86.8)
Negative predictive value	100% (64.6–100)
Accuracy	80.3%
AUC	0.684 (0.573–0.796)
Fisher's exact test	$p < 0.001$

Supplementary Table S4. Presence versus absence of pathological [^{18}F]FET uptake as a binary classifier, one scan per patient. 95% CI by the Wilson method. As in the primary analysis, no false-negative case occurred; the resulting 100% sensitivity and NPV are subject to the verification bias discussed in the main text.
